# Effects of demand-feeding and dietary protein level on nitrogen metabolism and symbiont dinitrogen gas production of common carp (*Cyprinus carpio*, L.)

**DOI:** 10.3389/fphys.2023.1111404

**Published:** 2023-02-07

**Authors:** Wouter Mes, Philippe Kersten, Roel M. Maas, Ep H. Eding, Mike S. M. Jetten, Henk Siepel, Sebastian Lücker, Marnix Gorissen, Maartje A. H. J. Van Kessel

**Affiliations:** ^1^ Department of Animal Ecology and Physiology, Radboud Institute for Biological and Ecological Sciences, Radboud University, Nijmegen, Netherlands; ^2^ Department of Microbiology, Radboud Institute for Biological and Ecological Sciences, Radboud University, Nijmegen, Netherlands; ^3^ Aquaculture and Fisheries Group, Wageningen University and Research, Wageningen, Netherlands

**Keywords:** ammonia, nitrogen cycle bacteria, gill, glutamate dehydrogenase (GDH), rhesus glycoprotein, symbiosis

## Abstract

Ammonia accumulation is a major challenge in intensive aquaculture, where fish are fed protein-rich diets in large rations, resulting in increased ammonia production when amino acids are metabolized as energy source. Ammonia is primarily excreted *via* the gills, which have been found to harbor nitrogen-cycle bacteria that convert ammonia into dinitrogen gas (N_2_) and therefore present a potential *in situ* detoxifying mechanism. Here, we determined the impact of feeding strategies (demand-feeding and batch-feeding) with two dietary protein levels on growth, nitrogen excretion, and nitrogen metabolism in common carp (*Cyprinus carpio*, L.) in a 3-week feeding experiment. Demand-fed fish exhibited significantly higher growth rates, though with lower feed efficiency. When corrected for feed intake, nitrogen excretion was not impacted by feeding strategy or dietary protein, but demand-fed fish had significantly more nitrogen unaccounted for in the nitrogen balance and less retained nitrogen. N_2_ production of individual fish was measured in all experimental groups, and production rates were in the same order of magnitude as the amount of nitrogen unaccounted for, thus potentially explaining the missing nitrogen in the balance. N_2_ production by carp was also observed when groups of fish were kept in metabolic chambers. Demand feeding furthermore caused a significant increase in hepatic glutamate dehydrogenase activities, indicating elevated ammonia production. However, branchial ammonia transporter expression levels in these animals were stable or decreased. Together, our results suggest that feeding strategy impacts fish growth and nitrogen metabolism, and that conversion of ammonia to N_2_ by nitrogen cycle bacteria in the gills may explain the unaccounted nitrogen in the balance.

## Introduction

Nitrogen accumulation, mostly in the form of ammonia, is a widespread problem in both natural and man-made aquatic systems such as aquaculture. For intensive aquaculture systems, this presents a major concern: the high stocking density at which animals are kept and the protein-rich feed that is provided in large rations to enhance growth results in high ammonia excretion (Crab et al., 2007; Bucking, 2017). Ammonia is the primary nitrogenous waste in most adult teleostean fish, although urea is also excreted in lower amounts (Sinha et al., 2016; Bucking, 2017). Ammonia, in particular the unionized form, is toxic to fish since it causes disruption of electrochemical gradients in the central nervous system and interference with cellular metabolism (Ip and Chew, 2010). High ammonia levels can additionally inhibit feed intake and growth, lead to anemia, oxidative stress, decreased welfare, and increased mortality (Ip et al., 2001; Ip and Chew, 2010; Schram et al., 2010; Yang et al., 2010; Li et al., 2020).

Fish produce ammonia as a result of amino acid catabolism. Dietary protein is first broken down to amino acids, which are absorbed in the gut and utilized for either protein retention (*i.e.,* growth) or converted into metabolic fuel. The latter occurs primarily in the fish liver through oxidative transdeamination, with alanine aminotransferase (ALT) and glutamate dehydrogenase (GDH) as two of the central enzymes involved (Ballantyne, 2001). Although the liver is the main organ where this process occurs, GDH activity has been measured in other fish organs as well, including the intestinal tract (Turner and Bucking, 2019).

The ammonia produced by GDH activity enters the bloodstream and is quickly excreted, primarily *via* the gills (∼90% of ammonia) (Bucking, 2017). While the exact mechanisms of ammonia excretion are not completely clear, it is known that rhesus glycoproteins play a role as ammonia transporters (Nakada et al., 2007; Nawata et al., 2007), which also increase in transcriptional level after a meal (Zimmer et al., 2010).

The amount of ammonia produced and excreted depends, among other factors, on the amount of feed consumed (Engin and Carter, 2001; Guo et al., 2012). In several fish species, a higher dietary protein content was correlated with higher hepatic GDH and ALT activities (Bucking, 2017), indicating an increase in ammonia production with increased dietary protein content.

In a recent study it was found that carp and zebrafish harbor nitrogen-cycle bacteria inside their gill cells that may alleviate the ammonia excretion resulting from feeding (van Kessel et al., 2016). In both species, ammonia-oxidizing *Nitrosomonas* and denitrifying bacteria were present in the gills and together were able to produce dinitrogen gas (N_2_), apparently despite the prevailing oxic conditions. The amount of N_2_ produced increased significantly when fish were fed, suggesting a relationship with dietary ammonia production. Additionally, the same study found that carp excreted less ammonia when fed on demand compared to those that were fed the same ration twice daily by hand. Combined with the increased growth of carp controlling their own feed intake (Klaren et al., 2013), these findings indicate favorable effects of such ‘demand-feeding’ on carp growth and nitrogen excretion. It was hypothesized by van Kessel et al. (2016), that the reduced ammonia excretion was the result of increased N_2_ production by the nitrogen cycle bacteria in the carp gills due to continuous availability of ammonia, but this was not further investigated. As of yet, the effect of the nitrogen cycle bacteria on the nitrogen balance (the amount of nitrogen consumed vs. the amount of nitrogen assimilated and excreted as ammonia or urea) of fish is unknown, while the implications of this symbiosis can be considerable for fish in both wild and cultured conditions. If a significant amount of ammonia is converted to N_2_ by symbionts, this would present a novel strategy to mitigate ammonia toxicity and could reduce the nitrogen load in aquaculture conditions.

These findings prompted us to study the effect of demand-feeding on the nitrogen metabolism of carp in more detail. In this study, the feeding protocol described in Klaren et al. (2013) was used with two different dietary protein levels. For both feed types, we compared the growth and nitrogen excretions of demand-fed carp with ones that were fed a similar ration twice a day by hand. N_2_ production of individual fish was measured at the end of the feeding experiment, and we assessed the activity levels of hepatic and intestinal enzymes related to ammonia production, as well as ammonia transporter gene expression in the gills. N_2_ production by carp was additionally measured in metabolic chambers. Based on the previous study, we hypothesized that growth efficiency would be improved by demand-feeding at both dietary protein levels (Klaren et al., 2013). Demand-feeding was predicted to have a significant impact on the nitrogen balance, with reduced ammonia excretion and increased N_2_ production rates per unit of feed intake. Additionally, fish fed a lower dietary protein content are also hypothesized to have a decreased GDH activity and ammonia excretion, while higher dietary protein content would lead to increased GDH activity and ammonia excretion (Guo et al., 2012; Bucking, 2017).

## Methods

### Experimental animals and diets

The carp feeding experiments and individual N_2_ production measurements were performed at the Radboud University in Nijmegen. Animal experiments were approved by the ethical committee of Radboud University (DEC 2019-0018, AVD1030020198606). The N_2_ production measurements of group-housed carp was performed at the Wageningen University and classified as not being an animal experiment according to Dutch legislation. All procedures applied to the animals were in line with the Dutch legislation (Act on Animal Experiments).

Juvenile common carp of both sexes (*Cyprinus carpio*, L., strain R3xF8, *n* = 120, mean body mass 16 g ± 10 g SD) were obtained from the hatchery at Wageningen University and Research Center (Wageningen, the Netherlands) and kept in Nijmegen tap water in recirculating systems at the animal facility of Radboud University (Nijmegen, the Netherlands) until the start of the experiments. Animals were fed commercial diet (Stella 2p, Skretting/Nutreco, Amersfoort, the Netherlands) at 1% BW per day. Three weeks before the start of the experiments, fish were tagged with passive integrated transponder (PIT) tags under light anesthesia (70 mg L^−1^ MS-222, pH 7.4), to allow individual identification. The tag was placed intramuscular, directly below the dorsal fin. Tag numbers were read with a PIT tag reader (Non-atec™, Lutronic International, Rodange, Luxembourg) and logged, after which the animals were transferred back to their tanks. A week before each replicate experiment (*N* = 3), 40 animals were weighed and divided over four experimental tanks (*n* = 10 per tank; mean total body mass of fish per tank for replicate 1: 234 g ± 5 g, replicate 2: 223 g ± 2 g, replicate 3: 278 g ± 8 g). Fish were allowed to acclimate to the new tanks for 1 week.

Two approximately iso-energetic and isolipidic experimental diets were formulated with 39% and 48% crude protein, respectively (based on Yun et al. (2015). Feed was produced by Research Diet Services (Research Diet Services B.V., Wijk bij Duurstede, the Netherlands; diet composition in Table 1). Proximate analysis of the feed was performed at the Wageningen University according to the protocol described in Maas et al. (2021).

**TABLE 1:** Composition of experimental diets used in the feeding experiment.

Ingredients	39% protein diet (g/kg ww)	48% protein diet (g/kg ww)
Fishmeal	251	340
Soybean meal	181	181
Full-fat extruded soybean	50	30
Fish hydrolysate	25	25
Brewer’s yeast	50	50
Wheat flour	166	166
Wheat middling	153	99
Hemoglobin powder	15	54
Lecithin	5	5
Fish oil	10	10
Soybean oil	10	10
a-cellulose	55	0
Ca(H_2_PO_4_)_2_	20	20
Vitamin/mineral premix*	10	10
Analysed nutrient content		
Dry matter	938	949
Crude protein	392	479
Crude lipid	55	53
Crude ash	88	100
Gross energy (MJ/kg)	19	19

^a^ Vitamin/mineral premix composition (mg kg^−1^ feed): iron (as FeSO_4_·7H_2_O), 50; zinc (as ZnSO_4_·7H_2_O), 100; cobalt (as CoSO_4_·7H_2_O), 2.4; copper (as CuSO_4_·5H_2_O), five; selenium (as Na_2_SeO_3_), 1; manganese (as MnSO_4_·4H_2_O), 25; magnesium (as MgSO_4_·7H_2_O), 300; chromium (as CrCl_3_·6H_2_O), one; iodine (as CaIO_3_·6H_2_O), 5. Vitamin premix composition (mg kg^−1^ feed): thiamin, 30; riboflavin, 30; nicotinic acid, 200; pantothenic acid, 100; pyridoxine, 30; cyanocaobalamin, 0.05; ascorbic acid, 500; alpha-tocopheryl acetate, 200 IU; folic acid, 15; retinylacetate, 15,000 IU; cholecalciferol, 2000 IU; menadione nicotinamide bisulphite (51%), eight; inositol, 200; choline (as choline chloride), 1,000; anti-oxidant BHT (E300-321), 100; calcium propionate, 1,000.

### Experimental tanks and demand-feeding device

Experimental tanks were set up in a climate-controlled room with a 12 h light and 12 h dark circadian rhythm (6:30 h lights on, 18:30 h lights off) at 22°C. Tanks were equal in volume and size (50 L water, length 40 cm x width 30 cm x height 50 cm) and all connected in parallel to the same biofilter. Water inflow was set at 1.5 L min^−1^, and water temperature was kept constant between 21°C and 23°C. The experimental tanks were placed at an 11°angle to remove excess feed and feces through the tank outflow. For each replicate, the treatment given to fish in a given tank was randomized.

For demand-fed groups, we used the experimental setup developed to study feed intake of carp by Klaren et al. (2013). In short, a pendulum was placed in the demand-fed tanks at 10 cm below the surface and a pendulum-controlled automated feeder was used to dispense feed into the tank when the pendulum was touched by the fish. The automatic feeders provided portions of on average 0.4 g ± 0.3 g (48% protein feed) and 0.5 g ± 0.4 g (39% protein feed). Carp were allowed to feed every 2 min by operating the pendulum. The number of activations per 24 h and amount of food (in grams) dispensed were calculated and corrected by the amount of uneaten feed that was collected from the outflow of the tank twice per day. Batch-fed groups were fed the amount of feed eaten by the demand-fed group on the previous day, divided into two equal portions given at 9:00 and 16:30 h. Batch-fed fish were allowed to feed for 30 min, after which remaining feed was removed and counted to determine the feed intake.

### Feeding experiment

**FIGURE 1:**
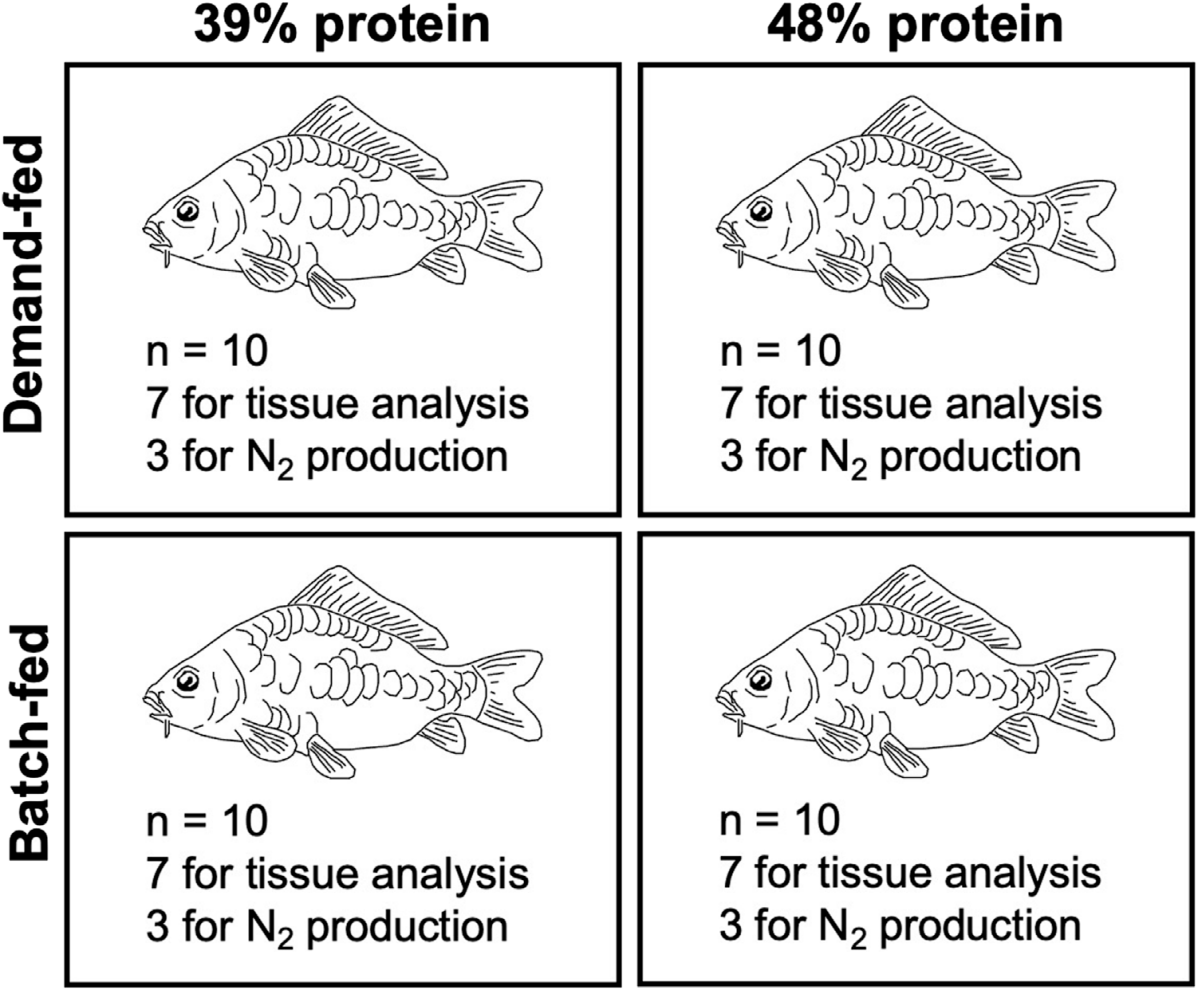
Experimental treatments in the carp feeding experiment and endpoint measurements. Schematic depiction of experimental groups of carp and treatments. Numbers of fish used for N_2_ measurements and tissue analysis are also provided. The feed experiment was performed in triplicate (*N* = 3).

Four groups of ten carp each were subjected to the feeding regimes for 21 days (Figure 1). Two groups were demand-fed, the other two batch-fed. One demand-fed group of carp was fed the 48% protein diet, whereas the other received the 39% protein diet. This feed experiment was repeated three times (*N* = 3, 12 tanks in total). The batch-fed groups received the same diet as their respective demand-fed peers. Feed intake was recorded daily. Additionally, on 5 days (day 4, 8, 11, 15 and 18 of the experiment) ammonia and urea excretion in each tank was measured. After 21 days, three fish from each tank were used for individual N_2_ production measurements. At the end of each experiment, all fish were euthanized, weighed, and sampled (Figure 1). From the initial weight, feed intake and final weight, we calculated absolute growth, specific growth rates, feed conversion ratios and survival as described elsewhere (Yadav et al., 2020). As fish were tagged, we were able to calculate growth and specific growth rates (SGR) for individual fish. Daily growth and SGRs were calculated based on the increase of body weight (w) using the following formulas:

$$Growth\left(g\;per\;day\right)=\left(W_{final}-W_{initial}\right)/number\;of\;days$$

$$SGR\left(\%\;body\;mass\;gained\;per\;day\right)=\left(\ln\left(W_{final}\right)-\ln\left(W_{initial}\right)\right)/number\;of\;days*100$$

The feed conversion ratio was calculated for each tank using the following formula:

FCR=Total feed dry matter intake/total wet weight gain

In the first replicate, four animals were lost during the experiment, other replicates had no mortalities during the experiment. For these animals, no SGR was calculated and FCRs were corrected for the mortality. A technical issue on day 18 of the first experiment caused a water temperature drop to 18°C in all tanks for less than 1 day before returning to normal.

### Nitrogen balance measurements

The nitrogen balance (nitrogen intake vs. nitrogen excretion and assimilation) per fish in each tank was calculated on 5 days during the feed experiments (on day 4, 8, 11, 15 and 18) over a period of 8 h. In order to compare nitrogen balances between demand-fed and batch-fed fish in the 8-h measuring period, we needed to correct for different timing of feed intake. We calculated feed intake relevant to the 8-h measuring period based on the relationship between feed intake and nitrogen excretion described by Chakraborty et al. (1992), *i.e.*, we assumed that ammonia excretion in carp followed the same dynamics as described, with all ammonia excreted as result of feed intake occurring within 22 h and so only part of the feed intake is relevant for the 8-h nitrogen balance. We then measured ammonia and urea excretions in each tank and estimated nitrogen retention. Finally, the amount of nitrogen unaccounted for by excretion and retention was calculated.

Gross nitrogen intake (GNI) was calculated as follows: feed intake (in grams of dry matter) relevant for the measurement period was calculated from the feed dispensed in each tank minus the uneaten feed collected. Protein intake was then calculated by multiplying feed intake with the respective dietary protein content. From the protein intake, we calculated the GNI with the Jones protein conversion factor (6.25). Since feces could not be collected, we did not measure fecal nitrogen loss and assumed equal protein digestibility between groups of 93%, similar to recent studies in carp that found minimal effects of feeding level on protein digestibility (Phan et al., 2019; Prabhu et al., 2019). Hence, digestible nitrogen intake (DNI) was calculated by multiplying GNI with the estimated digestibility. Fecal nitrogen losses (FN) were defined as the difference between GNI and DNI.

Ammonium concentrations were measured in each tank hourly from 9:00 (*t* = 0) to 17:00 (*t* = 8). For this, the water inflow was stopped, and 2 mL water samples were taken every hour. Samples were centrifuged at 15,000 rpm for 3 min and the supernatant was stored at −20°C until further analysis. Ammonium concentrations were measured using the orthophthaldialdehyde (OPA) method (Taylor et al., 1974), with the reagent volume modified for use in a 96-well plate (200 µL OPA reagent and 10 µL sample). The accumulation of ammonia from *t* = 0 to *t* = 8 h was calculated for each tank and the amount of nitrogen excreted as ammonia in 8 h was calculated. We controlled for possible removal of ammonia by bacterial biofilms in the tanks, by measuring the concentration of ammonia in a control tank without fish to which 100 µM NH_4_Cl was added. The ammonia concentration did not change over a period of 8 h.

Urea determination in water samples was performed with a urease treatment of the water samples, after which additional ammonium was measured using the OPA method described above. Urease suspension (90 μL, 6 mg mL^−1^ in dH_2_O) was added to 90 µL of the water sample and incubated at 30°C for 20 min, after which ammonium was measured as described above. The accumulation of urea from *t* = 0 to *t* = 8 h was calculated for each tank to determine the amount of nitrogen excreted as urea.

The retained nitrogen (RN) was calculated per group of fish for each of the five measuring days per experiment based on the predicted growth resulting from the feed intake and feed conversion ratios of each group and assumed stable moisture content (75% wet weight gain) and body protein content (70% dry weight gain) between groups.

Excreted nitrogen (Branchial and urinary nitrogen, BUN-measured) was calculated as the measured ammonia-N and urea-N excretion. Nitrogen unaccounted (UN) for was calculated as the difference between GNI and the sum of FN, RN and BUN-measured. To compare BUN-measured in the water to calculated BUN losses (BUN-calculated), BUN-calculated was calculated as the difference between GNI and the sum of FN and RN.

The nitrogen balance (the amount of nitrogen accounted for by assimilation and excretion) was calculated in two ways. First, the balance was calculated in absolute terms as nitrogen consumed and utilized: GNI=FN+RN+EN+UN

Second, the different fates of nitrogen in the balance were calculated as percentages of GNI.

The calculations were performed on 5 days per feeding experiment, for a total of 15 days for all 3 replicate experiments.

### Dinitrogen gas production of individual carp

N_2_ production of individual carp was measured according to the protocol described in van Kessel et al. (2016). The production of N_2_ was measured at the end of each feeding experiment, but measurements from the first replicate were unsuccessful and thus not used in the analysis. From the remaining tanks, 3 carp were used for N_2_ measurements, equaling six fish per treatment. Individual fish were transferred at 10:00 in the morning (1 h after feeding in case of batch-fed carp) into separate 5.5-L tanks filled with 4 L system water containing a closed gas phase, from which gas composition could be measured through rubber septa. After closing the tanks, the gas phase above the water was replaced by argon for 2 × 30 s to remove atmospheric N_2_. Subsequently, O_2_ was added to the gas phase to reach a final concentration of 20% (v/v). A control bottle with tank water containing no fish was used to assess N_2_ degassing from the water after flushing. Fish were kept in these tanks for 2.5 h and gas samples were taken every 20 min. Gas samples were analyzed with GC-MS (Agilent 5975c quadruple inert MS coupled to an Agilent 6890 gas chromatograph).

### Dinitrogen gas production of group-housed carp

To measure N_2_ production in an aquaculture setting, 40 common carp were divided over two 220 L metabolic chambers (90 × 60 × 45 cm) (total weight in tank 1: 1640 g, total weight in tank 2: 1730 g) and fed rations of approximately 1% body mass per day (Wageningen University, NL). As controls, two chambers without fish were used, one with tank water only and one supplemented with 100 µM NH_4_Cl. The chambers were sealed air-tight with lids and water locks. The air in the chambers was exchanged with a mixture of argon and oxygen (80%/20%) by flushing the chamber (8 L min^-1^, 1 h). After the removal of air, the gas phase in the chamber was circulated to secure dissolved oxygen levels (>4 mg L^−1^) in the water. As a control for leakages, 20 mL methane (CH_4_) was added to the gas phase of all chambers. 5 mL gas samples were taken every 15 min and water samples every 30 min for 240 min. Gas samples were analyzed with GC-MS as described above. CH_4_ was measured on a gas chromatograph equipped with a Porapak Q-column (100/120 mesh) and a flame ionisation detector (HP 5890 series II; Agilent Technologies, Santa Clara, CA, United States) with duplicate 100 µL injections. The ratio between N_2_ and CH_4_ was calculated for each chamber and normalized relative to the starting ratio.

### Sampling of carp

Carp from the feeding experiment were euthanized with an overdose of MS-222 (pH 7.0) and their weight and length were recorded. Subsequently, the gills from each animal were removed aseptically. Gill arches were collected separately, snap-frozen in liquid nitrogen and stored at −80°C for RNA isolation. Liver samples were snap-frozen and stored at −80°C for hepatic enzyme analyses and RNA isolation. The intestines were removed, and the proximal intestine was sampled. Total gut content was removed by squeezing the dissected gut from the distal to proximal end. Proximal intestines (1 cm section) were snap-frozen for enzymatic assays.

### Enzymatic assays

Glutamate dehydrogenase (GDH) activity was measured in livers and proximal intestines of carp with a commercially available kit (Sigma-Aldrich, St. Louis, MI, United States) according to manufacturer’s recommendations. Liver and proximal intestine samples were homogenized in the assay buffer (1 mg per 5 µL assay buffer) with a bead-beater (Retsch GmbH, Haan, Germany) run for 1 (liver) or 2 min (proximal intestine) at 30 Hz using 6 mm glass beads. Homogenates were diluted 100× and a 5 µL (liver) or 25 µL (proximal intestine) sample was used for the enzyme assays, which were performed in duplicate at 23°C. Activities (in milliUnits mg tissue^−1^, mU mg^-1^) were calculated according to the manufacturer’s instructions.

Similarly, alanine aminotransferase (ALT) activity was measured in carp livers using a commercially available kit (Sigma-Aldrich, St. Louis, MI, United States of America). Liver samples were homogenized in the assay buffer (1 mg per 5 µL buffer) by bead-beating as described above. Homogenates were diluted 100× and 20 µL sample was used for the enzyme assay, which was performed in duplicate at 25°C. Activities (mU mg^−1^) were calculated according to the manufacturer’s instructions.

### RNA isolation and real-time quantitative PCR

Total RNA was extracted from gill arches using TRIzol (Invitrogen, Carlsbad, CA, United States) reagent according to manufacturer’s protocol with an adjusted TRIzol volume (400 µL) and a second precipitation in sodium acetate and ethanol.

After removal of residual genomic DNA by treatment with RNAse-free DNase (BioRad, Hercules, CA, United States), 500 ng of RNA was reverse transcribed with iScript reverse transcriptase (BioRad, Hercules, CA, United States) in a 20 µL mixture containing iScript Reaction Mix and RNAse I inhibitor for 5 min at 25°C, 20 min at 46°C and 1 min at 96°C. After reverse transcription, cDNA samples were diluted with 140 µL ultra-pure H_2_O and 4 µL were used for qPCR reactions.

Relative expression of rhesus glycoprotein genes (*rhbg*, *rhcg-a* and *rhcg-b*) was measured with real-time quantitative PCR (RT-qPCR). In short, 4 μL of the cDNA was used as template in a reaction with 10 µL SYBR Green Master Mix (Biorad, Hercules, CA, United States), 0.4 µM forward and 0.4 µM reverse primers (Table 2) and ultrapure H_2_O in a total reaction volume of 20 µL. RT-qPCR was carried out using a CFX 96 qPCR machine (Biorad, Hercules, CA, United States) with the following conditions: 3 min at 95°C, followed by 40 cycles of 15 s at 95°C and 1 min at 60°C. All expression data was normalized to the mean expression of two reference genes (*β-actin* and 40S ribosomal protein S11 (RPS11)) (Vandesompele et al., 2002).

**TABLE 2:** Primer sequences used for gene expression studies of carp gills.

*Primer name*	*Primer sequence*	Accession number	*Reference*
Rhbg *F*	TCC​CAG​TTT​CCA​GGA​TGT​T	JX570877.1	Sinha et al. (2013)
Rhbg *R*	TGG​AAA​AAG​CCC​TGC​ATA​AG		
Rhcga *F*	ATC​CTG​AAC​ATC​CTC​CAT​GC	JX570878.1	Sinha et al. (2013)
Rhcga *R*	AAC​TTG​GCC​AGA​ACA​TCC​AC		
Rhcgb *F*	CAC​AAA​GCC​ACA​CAC​AGT​CC	JX570879.1	Sinha et al. (2013)
Rhcgb *R*	TCT​TTT​TCT​CGC​CGT​TCT​TG		
β-actin *F*	CAA​CAG​GGA​AAA​GAT​GAC​ACA​G	M24113.1	Huising et al. (2006)
β-actin *R*	GGG​ACA​GCA​CAG​CCT​GGA​T		
RPS11 *F*	CCG​TGG​GTG​ACA​TCG​TTA​CA	AB012087.1	Forlenza et al. (2008)
RPS11 *R*	TCA​GGA​CAT​TGA​ACC​TCA​CTG​TCT		

### Statistics

Statistical analyses were carried out using Graphpad Prism version 9.1 for Mac (Graphpad software, La Jolla, United States). Testing for normal distribution of data was done using the D’Agostino-Pearson test. If data were normally distributed, parametric statistical tests were used for further analyses. If data were not normally distributed, lognormality was tested and in case the data were log-normally distributed, parametric statistical tests were performed on the log-transformed data. The ratios of nitrogen accounted for in the experiment were transformed using a reciprocal transformation to obtain normally distributed data.

Two-way ANOVAs were used to test for statistical differences between experimental groups (with feeding type [demand-fed vs. batch-fed] and dietary protein level [48% protein vs. 39% protein diet] as factors). Post-hoc tests were performed in case of significant interaction between the two factors, using the Tukey test. Pearson correlation coefficients (*r*) of N_2_ production and hepatic GDH activity with growth parameters (absolute body weight and SGR) were calculated and tested for significance. For N_2_ measurements in the metabolic chambers, an F-test was used to test if the slopes of N_2_/CH_4_ ratios were significantly different from zero.

## Results

### Effects of feeding strategies on growth

We measured the growth and nitrogen metabolism of carp that were fed *ad libitum* for 21 days through a demand-feeding apparatus (demand-fed) or by hand (batch-fed), with diets that had a protein content of 48% or 39%. Carp that were demand-fed learned to operate the pendulum from day 1 onwards, as was previously observed (Klaren et al., 2013). The fish operated the pendulum with a circadian rhythm (Supplementary Figure S1), with both groups activating the pendulum more often when the light was on (peaking between 7:00 and 9:00).

Demand-feeding at different dietary protein levels affected multiple growth parameters, as shown in Table 3. Demand-fed carp had a significantly higher feed intake throughout the experiment (F (1, 236) = 67.06, *p* < 0.001), while dietary protein content did not significantly affect feed intake (F (1, 236) = 0.4569, *p* = 0.4985). Batch-fed animals were provided the same amount of feed as the demand-fed animals and were unable to eat this ration in the allocated 30 min. Daily growth (in grams per day) and specific growth rates were significantly higher in demand-fed carp (F (1, 112) = 9.103, *p* = 0.0032 and F (1, 112) = 15.92, *p* = 0.0001, respectively). There was also a significant effect of dietary protein level on SGRs (F (1, 112) = 9.412, *p* = 0.0027), with higher SGRs in fish fed the 48% protein diet. SGRs of batch-fed carp that received the 39% protein diet were lowest of all groups. Feed conversion ratios were higher in demand-fed carp, indicating a reduced growth efficiency compared to batch-fed carp (F (1, 8) = 7.978, *p* = 0.0223). Taken together, demand-feeding increased both feed intake and growth, while dietary protein levels only affected SGR.

**TABLE 3:** Growth performances carp fed different dietary protein levels within batch-fed and demand-fed strategies in a 3-week feed experiment.

	*DF-P48*	*BF-P48*	*DF-P39*	*BF-P39*	*Pooled SD*	*Feeding strategy*	*Dietary protein level*
*Fish/tank*	10	10	10	10			
*Tanks*	3	3	3	3			
*Survival (%)*	100	90	96.7	100			
*Initial body weight (g)*	25.0	24.6	24.2	24.3	9.0	ns	ns
*Final body weight (g)*	56.9	51.6	54.1	46.5	18	ns	ns
*Growth (g/d)*	1.52	1.28	1.42	1.06	0.51	**	ns
*Feed intake (g DM/d)*	1.69	1.08	1.66	1.01	0.59	***	ns
*FCR*	1.07	0.89	1.13	0.91	0.14	*	ns
*SGR (%/d)*	3.97	3.68	3.80	3.06	0.70	***	**

DF: demand-fed, BF: batch-fed, P48: 48% protein diet, P39: 39% protein diet, pooled SD: pooled standard deviations, ns: not significant, DM: dry matter, FCR: feed conversion ratio, SGR: specific growth rate. Means are shown for each growth parameter, with body weight and growth rates calculated for individual fish (*n* = 116) and feed intake and FCR calculated for tanks of fish (*n* = 12). Significance of feeding strategy (DF vs. BF) and dietary protein level (P48 vs. P39) factors was tested in a two-way ANOVA and significance of factors on growth parameters is indicated using asterisks. * *p* < 0.05, ** *p* < 0.01, *** *p* < 0.001.

### Effects of feeding strategies on nitrogen balance of carp

On 5 days during the feeding experiment replicates, we determined the nitrogen balance of each tank by calculating the protein intake and growth of the carp in the tank and measuring ammonia and urea excretion. In Table 4, the average nitrogen balances of each diet and feeding strategy are shown. Gross nitrogen intake (GNI) was significantly higher in demand-fed carp [F (1, 55) = 15.05, *p* = 0.0003], reflecting the higher feed intake during the overall feed experiment. The retained nitrogen in demand-fed carp tanks was also significantly higher as a result of increased intake [F (1, 55) = 5.502, *p* = 0.0226]. Nitrogen excreted as ammonia and urea was affected by both feeding method and dietary protein content [F (1, 55) = 7.572, *p* = 0.008, F (1, 55) = 4.859, *p* = 0.0317, respectively]. In each group, there was nitrogen unaccounted for in the nitrogen balance. In absolute terms, the amount of nitrogen that was unaccounted for by growth or ammonia and urea excretion was significantly higher in demand-fed groups [F (1, 55) = 17.75, *p* < 0.0001]. The nitrogen unaccounted for resulted in a marked difference between calculated branchial and urinary nitrogen loss (BUN) and measured excreted nitrogen in the form of ammonia and urea.

**TABLE 4:** Nitrogen balances in demand-fed and batch-fed carp tanks at two dietary protein levels.

Nitrogen balance (mg/fish in 8 h measuring period)	*DF P48*	*BF P48*	*DF P39*	*BF P39*	*Pooled SD*	*Effect Feeding strategy*	*Effect Dietary protein*
*No. Tanks*	3	3	3	3			
*Measuring days/tank*	5	5	5	5			
*Gross N intake*	48	30	42	22	19	***	ns
*Digestible N*	45	27	39	21			
*Fecal N (estimated)*	3	2	3	2			
*Branchial and urinary N*	29	15	24	10	12	***	
*Retained N*	16	13	16	10	6	*	ns
*Excreted N*	9.1	5.8	6.2	5.5	3	**	*
*Unaccounted N*	20	9	17	5	11	***	ns

DF: demand-fed, BF: batch-fed, P48: 48% protein diet, P39: 39% protein diet, ns: not significant. All nitrogen balance parameters are expressed in mg nitrogen per fish in an 8-h measuring period. Values are mean values of all measuring periods (*n* = 60), with 15 measurements per combination of feeding strategy and dietary protein level. Significance of feeding strategy (DF vs. BF) and dietary protein level (P48 vs. P39) was tested with two-way ANOVAs for nitrogen balance parameters. The statistical significance of each factor is indicated using asterisks. * *p* < 0.05, ** *p* < 0.01, *** *p* < 0.001. Fecal N loss and digestible N were calculated using estimated protein digestibility and were thus constants and not tested.

**FIGURE 2:**
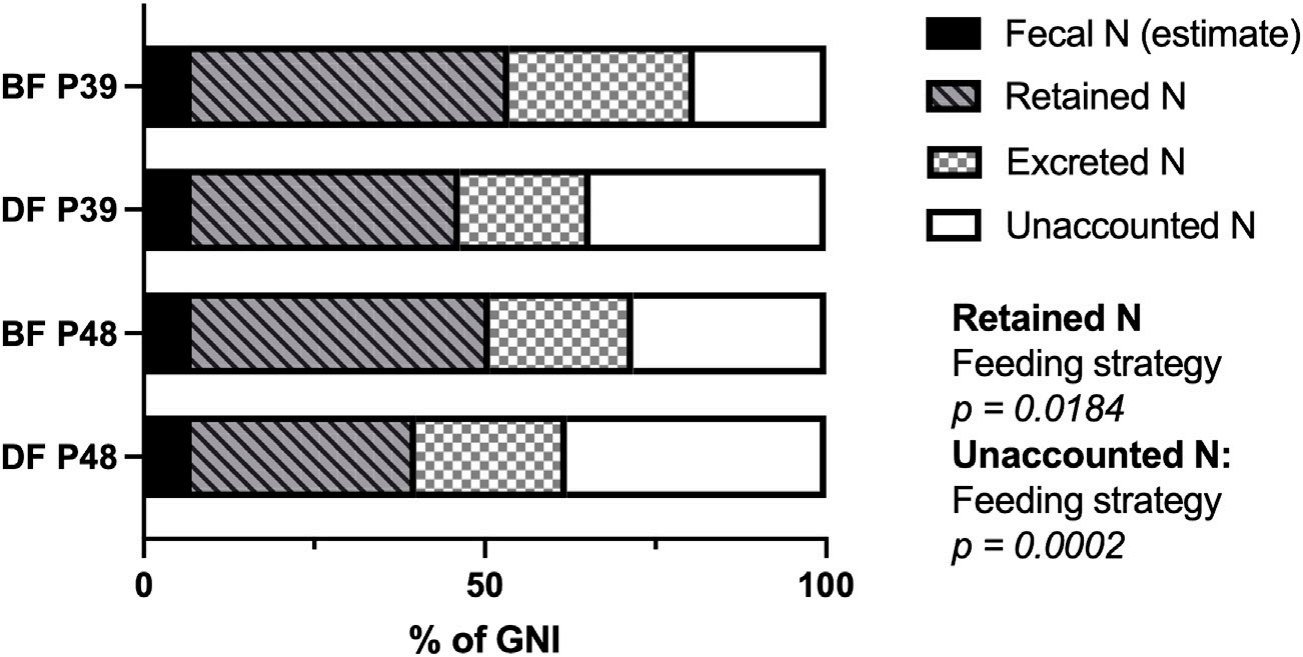
Nitrogen balances of demand-fed and batch-fed carp tanks fed at two dietary protein levels. The nitrogen (N) allocation during measuring days (*n* = 60) are expressed as percentages of gross nitrogen intake (GNI) for each combination of feeding strategy and dietary protein level. The combinations of feeding strategy (DF: demand-fed, BF: batch-fed) and dietary protein content (P39: 39% protein diet, P48: 48% protein diet) are indicated on the y-axis. Significance was tested with a two-way ANOVA for each nitrogen fraction (retained N, excreted N, unaccounted N) except fecal N which was not calculated and assumed as constant, with the factors “Feeding strategy” and “Dietary protein level” .Feeding strategy had a significant effect on the percentage of retained N (*p* = 0.0184) and the percentage of unaccounted N (*p* = 0.0002).

Since the GNI of demand-fed fish and batch-fed fish was significantly different, we also determined the nitrogen balance as a percentage of GNI. In Figure 2, the overall nitrogen balances in percentages of GNI are shown for each combination of feeding strategy and dietary protein level. When corrected for the differences in GNI, excreted nitrogen was similar between groups of carp (*p* > 0.05). In contrast, the nitrogen unaccounted for was affected by feeding strategy (F (1, 55) = 15.64, *p* = 0.0002) and retained nitrogen was affected by feeding strategy as well (F (1, 8) = 7.978, *p* = 0.0184). Specifically, demand-fed carp had a higher percentage of nitrogen unaccounted for, and retained a lower percentage of their nitrogen compared to batch-fed carp. Overall, the significant difference in GNI between demand-fed and batch-fed carp explains the differences in nitrogen excreted, but not the unaccounted nitrogen, which was also significantly different when corrected for GNI. The percentage of nitrogen retained by demand-fed fish was also reduced, which is in line with the significantly increased FCR in these animals.

### Dinitrogen gas production by carp

**FIGURE 3:**
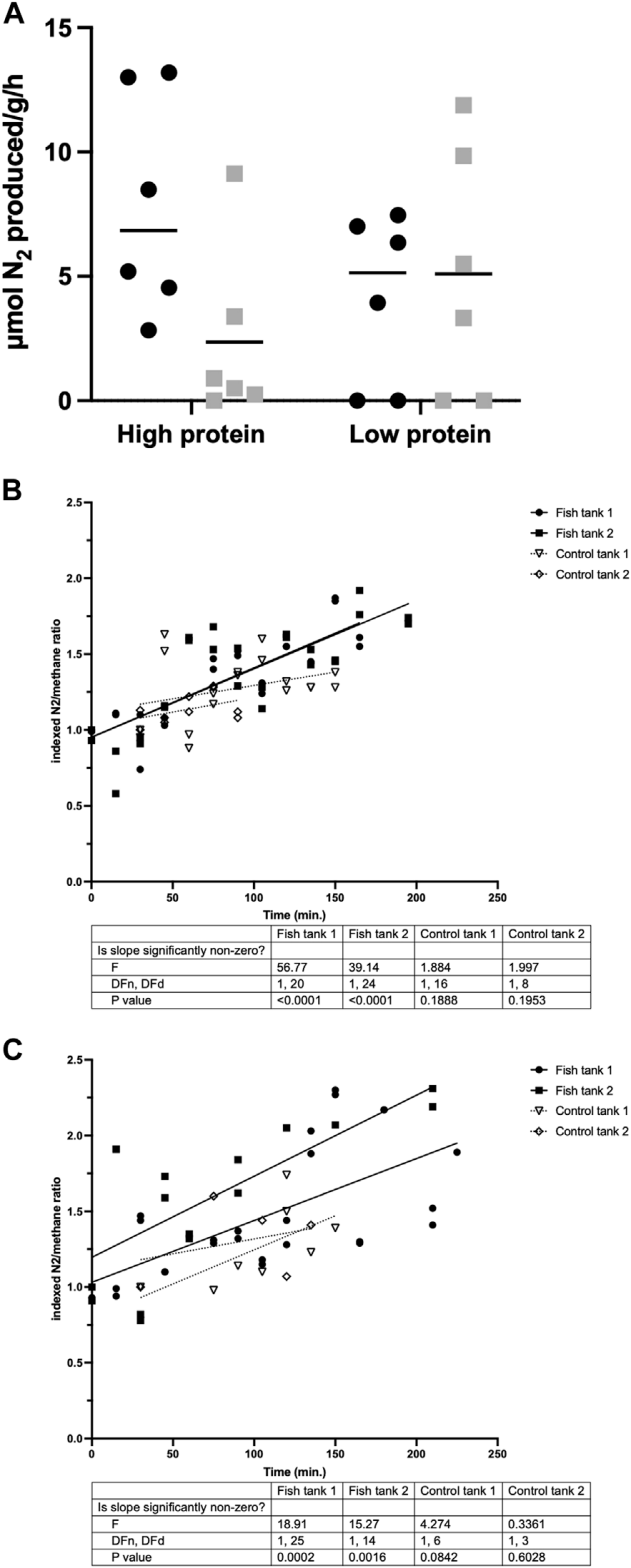
N_2_ production of individual and group-housed carp. **(A)** Individual N_2_ production (in µmol/g body weight/hour) over a period of 2.5 h was measured in individually housed fish (*n* = 6 per treatment). Demand-fed individuals are shown as black circles (•), batch-fed individuals as grey squares (■). Values shown are N_2_ production above increases in a control bottle without fish. If the value was below the increase in the control bottle, this is shown as 0 in the graph. Horizontal bars indicate median N_2_ production values. Significance was tested with a two-way ANOVA with the factors ‘Feeding strategy’ and ‘Dietary protein level’. **(B,C)** N_2_ production of group-housed carp (expressed as N_2_/CH_4_ ratios normalized to initial ratio) was measured in tanks containing 20 carp and in control tanks without fish. Duplicate values are shown for each timepoint. Simple linear regression analysis was performed to obtain the slope of N_2_ increase over time for each tank separately. F-tests were performed to test if the calculated slopes were significantly different from zero. Slopes of tanks containing fish are shown as solid lines, slopes of control tanks as dotted lines.

N_2_ production was measured for six individual carp per group (Figure 3A), in order to determine if the activity of nitrogen-cycle bacteria could explain the observed gap in the nitrogen balance. N_2_ production was observed within 2.5 h by carp in all experimental groups, with all tested carp from the demand-fed 48% protein diet group producing N_2_. Average rates of N_2_ production were highest in demand-fed carp fed the 48% protein diet (8 µmol per gram body weight per hour) and lowest in batch-fed carp fed the 48% protein diet (2 µmol per gram body weight per hour). With these rates of N_2_ production extrapolated to individual fish during 8 h, N_2_ production can account for 0.75–3.64 mmol N (10.4–51.0 mg N), which is in the same order of magnitude as the nitrogen unaccounted for in carp tanks (Table 4). No statistically significant differences were observed between groups, but there was a positive trend in N_2_ production in relation to body weight (*R* ^2^ = 0.14, *p* = 0.0695). N_2_ production by carp was additionally confirmed by housing fish in metabolic chambers and feeding the animals a single feed ration (Figures 3B,C). Here, we measured that in tanks with fish, N_2_ increased significantly over time (*p* < 0.01 for all measurements), while in control tanks without fish there was no significant increase (*p* > 0.05 for all measurements).

### Effects of feeding strategy on nitrogen metabolism and excretion

**FIGURE 4:**
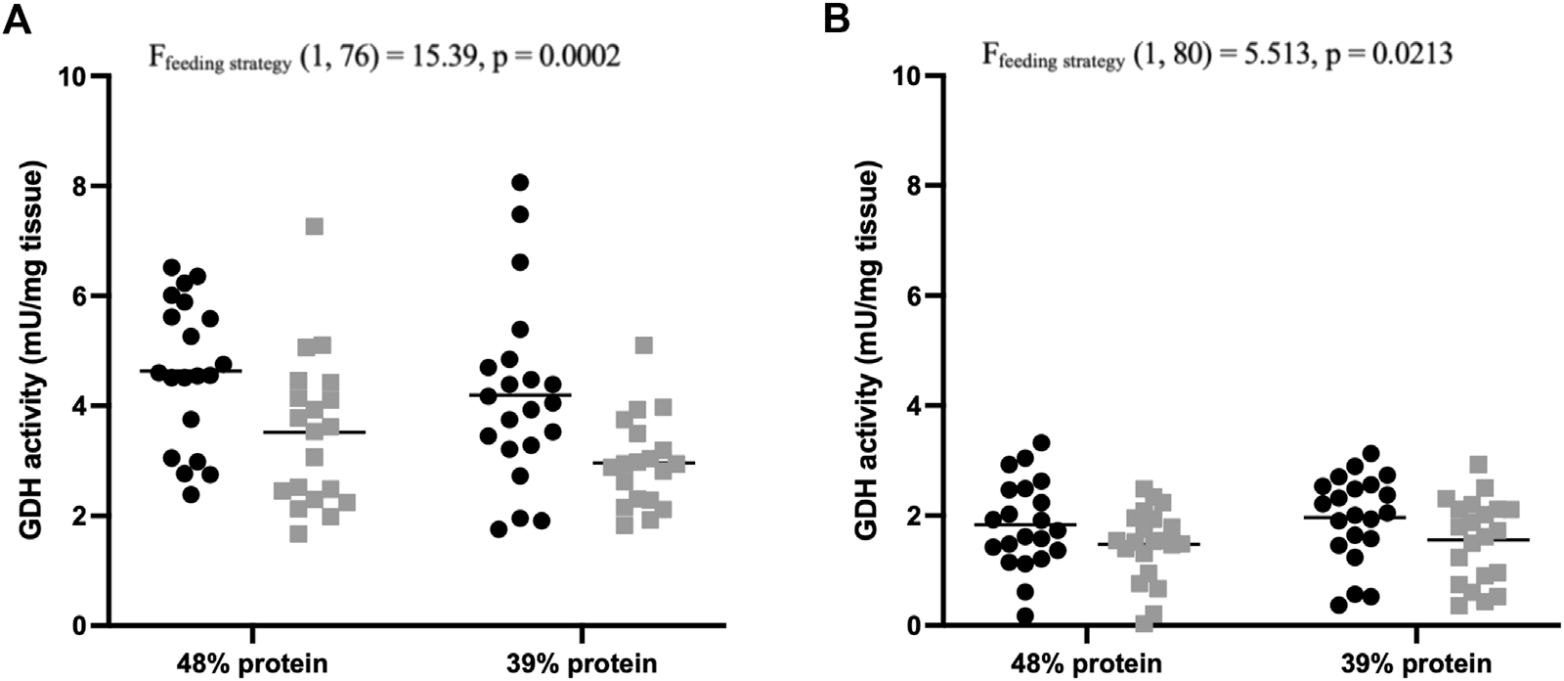
Glutamate dehydrogenase activity of carp liver and proximal intestines. **(A)** Hepatic GDH activities (mU/mg tissue) of demand-fed and batch-fed carp fed different dietary protein contents (*n* = 21 per treatment). Demand-fed individuals are shown as black circles (•), batch-fed individuals as grey squares (■). Each point represents the mean activity (from duplicate measurements) of a single carp. Horizontal bars indicate mean activity. Significance was tested with a two-way ANOVA with the factors ‘Feeding strategy’ and ‘Dietary protein’. Feeding type had a significant effect on hepatic GDH activity (*p* = 0.0002). **(B)** Intestinal GDH activities (mU/mg tissue) of demand-fed and batch-fed carp fed different dietary protein contents (*n* = 21 per treatment). Demand-fed individuals are shown as black circles (•), batch-fed individuals as grey squares (■). Each dot represents the mean activity (from duplicate measurements) of a single carp. Horizontal bars indicate mean activity. Significance was tested with a two-way ANOVA with the factors ‘Feeding strategy’ and ‘Dietary protein level’. Feeding strategy had a significant effect on intestinal GDH activity (*p* = 0.0213). Note that the y-axis is the same in both panels to make comparison between hepatic and intestinal GDH activities easier.

Following the feeding experiment, the nitrogen metabolism of individual carp was investigated by measuring activity of enzymes involved in amino acid catabolism in the liver and the proximal intestine. The activity of GDH was significantly affected by demand-feeding in both liver [F (1, 76) = 15.39, *p* = 0.0002; Figure 4A] and proximal intestine [F (1, 80) = 5.513, *p* = 0.0213; Figure 4B]. Hepatic GDH activity was also positively correlated with SGR (*R* ^2^ = 0.1143, *p* = 0.0022). The activity of ALT in liver samples was unaffected by demand-feeding or dietary protein content (data not shown).

**FIGURE 5:**
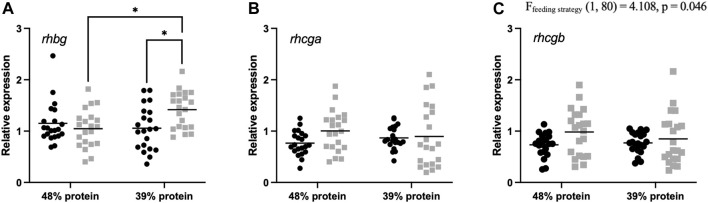
Rhesus glycoprotein transporter relative gene expression in gills of demand-fed and batch-fed carp fed 48% or 39% protein diets. Relative expression in demand-fed and batch-fed carp gills of **(A)** Rhesus glycoprotein B (rhbg), **(B)** Rhesus glycoprotein C type A (rhcg-a), and **(C)** Rhesus glycoprotein C type B (rhcg-b). Gene expression is shown as expression relative to *β*-actin and RPS11 expression. Gene expression was measured in 21 animals per group (*n* = 21). Demand-fed individuals are shown as black circles (•), batch-fed individuals as grey squares (■). Each dot represents one animal, horizontal bars indicate mean relative expression. Significance was tested with a two-way ANOVA with the factors ‘Feeding strategy’ and ‘Dietary protein level’. Feeding strategy had a significant effect on rhcg-b expression (*p* = 0.046). In case of significant interaction effects (rhbg), a Tukey multiple comparisons *post hoc* test was used. Asterisks indicate significant differences between groups based on *post hoc* tests, * *p* < 0.05.

Finally, we determined the expression levels of ammonia transporter genes. Branchial rhesus glycoprotein (*rhbg, rhcg-a* and *rhcg-b*) mRNA expression was assessed for individual demand- and batch-fed carp (Figure 5). Demand-fed carp had a significantly lower expression of *rhcg-b* compared to batch-fed individuals [F (1, 80) = 4.108, *p* = 0.046]. Transcript abundance of *rhbg* was increased in the batch-fed fish receiving the 39% protein diet compared to other groups (batch-fed, 48% protein diet [*q =* 4.377, *p* = 0.0141] and demand-fed, 39% protein diet [*q =* 4.264, *p* = 0.0177]). Expression levels of *rhcg-a* were unaffected by either factor. Thus, demand-feeding decreases relative *rhcg-b* expression at both dietary protein levels, whereas relative *rhbg* expression was significantly increased in batch-fed fish on a 39% protein diet.

## Discussion

In this study, we determined the effects of dietary protein level and demand-feeding vs. batch-feeding on growth and nitrogen metabolism in common carp. We determined the rates of ammonia, urea, and N_2_ excretion, the latter of which is produced by unique nitrogen-cycle bacteria in the gill epithelium of these animals. Together, our findings show that the amount of N_2_ produced by the fish-bacterial symbiosis contributes significantly to the nitrogen balance of fish in this experimental setup.

Carp that were allowed to feed themselves grew faster, but less efficiently than carp fed in batches (by hand). Thus, the increased growth rates are due to an increased feed intake in demand-fed fish. This is interesting, since Klaren et al. (2013) found that demand-fed carp of similar age and size grew faster *and* were more efficient in their growth than batch-fed carp, with comparable feed intake. While the experimental demand-feeding setup was comparable between studies, the diets used here were different in composition from that in Klaren et al. The difference in diet may have affected the feed efficiency of the fish. Additionally, the batch-fed carp in our experiment were not able to eat the same amount of feed as their demand-fed counterparts, which was not the case in the previous study.

Besides the effects of feeding strategy on the growth rates of carp, we observed marked changes in the nitrogen balances calculated for groups of carp. In our feeding experiment, we measured the nitrogen intake and excretion over periods of 8 h and calculated nitrogen retention. Due to a higher nitrogen intake in demand-fed animals, a result of the increased feed intake, the absolute amount of nitrogen accounted for by excretion and retention was increased in the demand-fed groups. However, when the nitrogen balances were corrected for differences in nitrogen intake, only the unaccounted fraction of nitrogen was increased in demand-fed fish. Nitrogen excretion was not affected, while nitrogen retention of demand-fed fish was lower than in batch-fed fish. The reduced nitrogen retention is in line with the overall lower feed efficiency found in demand-fed carp. On most days and in most tanks, there remained a “gap” in the nitrogen balance between nitrogen intake on the one hand, and nitrogen assimilated into growth and excreted on the other. Since this gap was significantly larger in demand-fed animals, there is an effect of feeding strategy on the overall nitrogen balance, similar to what was found by van Kessel et al. (2016).

In the nitrogen balance, the percentage of nitrogen that was unaccounted for by retention and excretion was on average 19%–38%, depending on the feeding strategy and dietary protein level. Several previous studies have reported gaps in the nitrogen balance of several fish species, and it thus seems that nitrogen balances cannot always be closed in fish (Lauff and Wood, 1996; Wood, 2001; Kajimura et al., 2004; Godoy-Olmos et al., 2022). Part of the nitrogen gap may be explained by increased fecal nitrogen loss, which we were unable to calculate for our animals as we could not distinguish between uneaten feed pellets and feces during the feeding experiment and was therefore estimated to be 7% based on previous studies in carp. However, previous literature on protein digestibility and fecal nitrogen loss in carp reported values in the range of 5%–10% of total nitrogen intake (Phan et al., 2019; Prabhu et al., 2019), which would translate to at most an additional 8 mg nitrogen in batch-fed groups (11% of missing nitrogen) and 14 mg in demand-fed groups (approximately 7% of missing nitrogen) in our experiment. We assumed comparable nitrogen excretion patterns between groups of fish, independent of the time of the day when fish were fed. Since diurnal variation in ammonia excretion patterns is not expected and has not been reported, it is unlikely that this explains the differences in unaccounted nitrogen. Other nitrogenous compounds (e.g., amino acids, mucus proteins) can also result in nitrogen loss, but have not been shown to comprise a large share of nitrogen excretion. This also did not explain all missing nitrogen in other fish species either, where 12%–20% nitrogen still remained unaccounted for after taking other nitrogenous compounds into account (Kajimura et al., 2004).

An as of yet unexplored mechanism to account for the missing nitrogen in the nitrogen balance is N_2_ production by symbiotic nitrogen-cycle bacteria in the gills of carp. We measured N_2_ production of individual animals after the feeding experiment, and production rates were in the same order of magnitude as the missing nitrogen (milligrams of N during an 8-h period). Although demand-feeding did not have a significant effect on N_2_ production rates at the end of the feeding experiment, N_2_ production showed a positive trend with body weight. The potential of N_2_ production was verified using a new batch of carp group housed (*N* = 20) in metabolic chambers. In this setup we were able to measure N_2_ production as well, thus confirming the ammonia converting activity into N_2_ gas of fish-associated symbiotic nitrogen cycle bacteria.

Increased GDH activity and decreased rhesus glycoprotein expression in the gills of carp are in agreement with N_2_ production as explanation for the gap in the nitrogen balance. Hepatic GDH activity is the main contributor to ammonia production in fish (Bucking, 2017) and an elevated GDH activity in demand-fed fish indicates an increase in ammonia production potential in these animals. GDH activity is also a key contributor to ATP production in the liver of other fish species (Li et al., 2020) and an increase in GDH activity thus indicates increased use of amino acids as energy source. This also can explain the increased FCR in demand-fed carp, since comparatively fewer amino acids are utilized for growth. Indeed, increased hepatic GDH activity is also correlated to reduced protein efficiency and increased ammonia excretion in rainbow trout (*Oncorhynchus mykiss*) (Martin et al., 2003; Betiku et al., 2018).

In contrast, the expression of rhesus glycoproteins in the gills of demand-fed carp remained stable (in the case of *rhbg* and *rhcg-a*) or was lower than in batch-fed animals (in the case of *rhcg-b* expression). Previous studies have shown increases in rhesus glycoprotein expression after feeding (Zimmer et al., 2010; Rodela et al., 2012), and while the specific mechanisms of ammonia transport are not well studied in carp, upregulation under high external ammonia suggests a similar involvement of rhesus glycoproteins (Sinha et al., 2013). Hence, a downregulation of expression in demand-fed carp can suggest a reduced reliance on rhesus-mediated ammonia transport. Alternatively, the lower expression of rhesus glycoproteins may be explained by a reduced ‘peak’ of ammonia in this feeding regime as feed intake is more spread out over the day compared to batch-fed carp. This was previously observed in demand-fed carp and seabream (van Kessel et al., 2016; Godoy-Olmos et al., 2022), although not in European eel (Heinsbroek et al., 2008). These results (increased activity of ammonia-producing enzymes and reduced transporter expression) fuel the hypothesis that a larger amount of the ammonia produced by amino acid oxidation is converted to N_2_ before being excreted into the surrounding water.

Our approach of tracking individual growth by PIT-tagging fish additionally allowed us to compare individual growth rates within the 3-week feeding experiment and to correlate enzyme activity and gene expression to the growth rates of individual fish. With this approach, it became clear that specific growth rates vary considerably within experimental groups, with some animals having a nearly 2-fold higher SGR than others in the same tank. Unfortunately, individual feed intake could not be measured in this setup and thus we do not know the extent to which differences in feed intake influenced the differences in SGR. In the case of hepatic GDH activities, we found a clear correlation with SGR, which would not have been noted when comparing the mean GDH activity per group only. A similar finding was reported by Walzem et al. (1991), in which hepatic enzyme activities of trout correlated linearly with individual growth rates. While the reason for this strong correlation is unknown, it can indicate an increased amino acid catabolism in fast-growing carp, and thus also an elevated ammonia production. Alternatively, the correlation may be explained by increased feed intake of fast-growing fish.

If N_2_ production by gill-associated bacteria indeed accounts for a significant part of the ‘gap’ in the nitrogen balance, this would have profound implications for research into the metabolic utilization of protein in fish. For instance, when estimating the proportion of amino acid catabolism from ammonia and urea excretion (as is done for example by Wang et al. (2021)), the true extent of amino acid turnover will be underestimated since part of the catabolized amino acids will not be measurable as ammonia or urea. In contrast, if nitrogen excretion is estimated based on the difference between digested nitrogen and retained nitrogen (Tran-Duy et al., 2008), the total ammonia excretion is overestimated. Similarly, when total nitrogen is measured using conventional methods (Kajimura et al., 2004), N_2_ will not be detected and thus does not add up to the ‘total’ nitrogen excreted by fish. Since studies often utilize these approaches to obtain estimates of protein utilization in fish (Lauff and Wood, 1996; Ferreira et al., 2019; Wang et al., 2021), to optimize feeding regimes and nutritionally balanced diets, it is key to have knowledge about the magnitude of N_2_ production in fish.

In our experimental setup, N_2_ production measurements in individual carp revealed several interesting aspects: while N_2_ production was not significantly affected by a 3-week period of different feeding strategies or dietary protein levels, differences were present between groups of fish. Of particular interest are the animals that did not produce measurable amounts of N_2_ at the end of the feeding experiment. This absence of N_2_ production in some fish can be explained in two ways: it is possible that fish without N_2_ production did not consume feed in the period before sampling and therefore were not producing ammonia at high rates, which is in agreement with the strongly reduced N_2_ production of starved carp reported previously by van Kessel et al. (2016). Alternatively, these particular fish might lack the nitrogen cycle bacteria required for N_2_ production. Whether all carp have the nitrogen cycle bacteria present in their gills and if and how they can obtain them is important for determining the potential impact of this symbiosis on the fish’s nitrogen excretion.

*Nitrosomonas* 16S rRNA sequences were also recovered from the gills of Atlantic salmon (*Salmo salar*), Yellowtail kingfish (*Seriola lalandi*), and Red snapper (*Lutjanus campechanus*) (Tarnecki et al., 2016; Legrand et al., 2018; Minich et al., 2020). It can be hypothesized that N_2_ production by bacteria also occurs in these fish species. Follow-up experiments where N_2_ production is measured in these fish species can determine the impact of N_2_ production by the gill microbiome on nitrogen excretion by fish more broadly.

Together, the results suggest that the feeding regime affects nitrogen balance and growth in carp. In all experimental groups, considerable amounts of nitrogen were unaccounted for in the nitrogen balance, and carp were shown to excrete N_2_ at a rate that is in the same order of magnitude as the unaccounted amounts of nitrogen. The N_2_ is most likely produced by symbiotic branchial nitrogen cycle bacteria. Since these bacteria are also found in other fish species, this phenomenon is likely to be a more universal feature and may have profound and as of yet unappreciated consequences for understanding the nitrogen balance in fish. It is therefore important to investigate this intriguing symbiosis in more detail, as it impacts our understanding of the role of fish and their microbiome from a functional perspective.

## Data Availability

The original contributions presented in the study are included in the article/Supplementary Materials, further inquiries can be directed to the corresponding authors.
