# Supplementary material for: Effects of demand-feeding and dietary protein level on nitrogen metabolism and symbiont dinitrogen gas production of common carp (Cyprinus carpio, L.)
Source: Front Physiol. 2023 Feb 7;14:1111404. doi: 10.3389/fphys.2023.1111404 (PMC9941540; doi:10.3389/fphys.2023.1111404)
Supplement: Supplementary file 1 [file Table1.DOCX]

**Supplementary figures**

***Fig. S1: Daily pattern of pendulum activations in demand-fed groups of carp fed different dietary protein content*** ***diets****. Pendulum activations lead to feed distribution and show a daily rhythm during the 21-day feeding experiment and thus show when demand-fed carp are feeding. Boxes show interquartile range and median activations and whiskers the 2.5-97.5 percentile values in a 2-hour interval over three 21-day feeding experiment. Dots indicate values outside 2.5-97.5 percentile.*
